# The influence of low-fidelity simulator training on canine peripheral venous puncture procedure

**DOI:** 10.14202/vetworld.2021.410-418

**Published:** 2021-02-15

**Authors:** Dayane Aparecida Francisco da Silva, Aline Angela Fernandes, Ana Evellyn Ventrone, Ariane Dias, Ana Maria Siqueira Silveira, Cecilia Laposy Santarém, Gabrielle Gomes dos Santos Ribeiro, Rosa Maria Barilli Nogueira

**Affiliations:** 1 Laboratory of Simulation and Skills Training in Veterinary Medicine, School of Agricultural Sciences, Universidade do Oeste Paulista, Presidente Prudente, Sao Paulo, Brazil; 2 Veterinary Medicine Undergraduate Program at Universidade do Oeste Paulista, Presidente Prudente, Sao Paulo, Brazil; 3 Department of Support and Diagnosis, Laboratory of Veterinary Clinical Pathology, Universidade do Oeste Paulista, Presidente Prudente, Sao Paulo, Brazil; 4 Department of Ph.D. Program in Pathophysiology and Animal Health, Universidade do Oeste Paulista, Presidente Prudente, Sao Paulo, Brazil; 5 Department of Statistical Analysis, Universidade do Oeste Paulista, Presidente Prudente, Sao Paulo, Brazil

**Keywords:** canine, clinical skills training, evaluation, self-confidence, veterinary simulation

## Abstract

**Background and Aim:**

Blood collection from dogs is the most commonly performed procedure in the medical clinic. However, different factors can interfere with the quality of the material collected, potentially causing complications for patients. Simulated skill training is a teaching strategy designed to provide early training to students, develop their skills and self-confidence, and increase the procedure’s success while reducing complications. Therefore, the aim of this study was to evaluate skill training using a low-fidelity simulator in the peripheral venipuncture procedure and examine the training’s influence on the *in vivo* procedure.

**Materials and Methods:**

To assess skill training, this study used a low-fidelity simulator in the peripheral venipuncture procedure and examines the training’s effect on the *in vivo* procedure. In total, 100 dogs, 65 undergraduate students, 3 veterinarians, and 4 previously trained evaluators participated. The canine *in vivo* venipuncture procedure was evaluated both before and after the simulated skill training and the low-fidelity simulator training. Data were collected on participants’ self-confidence levels.

**Results:**

Local complications occurred during *in vivo* practice; however, after training, they decreased. Gloves were more frequently used during the procedure, resulting in a reduction of both harvest attempts and complications, as well as increased levels of self-confidence in post-training participants. The simulator developed had low fidelity, low cost, and was easy to create.

**Conclusion:**

Skill training in peripheral venipuncture using a low-fidelity simulator positively influences student learning, increases their self-confidence during *in vivo* harvesting, and reduces the complications of the procedure, improving patient well-being.

## Introduction

The most common procedure performed on patients in the hospital environment is vascular access and blood collection for diagnostic or therapeutic purposes [1]. A professional with blood collection experience in the peripheral venipuncture procedure increases its precision before, during, and after material collection [2,3]. However, adjustments may occur owing to factors such as patient containment, antisepsis failure, phlebitis, hematomas, blood vessel rupture, and changes in cell morphology [2-4]. Collection methods used to obtain a blood sample from dogs involve either a syringe and needle or the vacuum tube system [4]. As these different protocols require the veterinarian to perform the entire procedure efficiently, training students in this procedure are essential [4,5]. Recently, in Brazil, the regulatory standard nº 38 of 17 April 2018, from the National Council for Animal Experimentation (CONCEA), declared that the use of live animals in demonstrative and observational classes is prohibited, indicating that it should be replaced by other teaching tools of sufficient quality to maintain and improve learning conditions [6].

Consequently, innovative technologies are being introduced in the areas of human and animal health, aiming to achieve an approximation of clinical routine and patient well-being [7]. Clinical simulation involves skills training and scenarios using dummies. These are essential instruments for the improvement of both trained graduates and professionals [7,8]. Simulations with mannequins can be classified according to the use of resources in skill training (part task trainer), such as a standardized patient, computer based, and high-fidelity mannequins [8,9]. Clinical simulations recreate a real situation in an artificial environment with the aim of learning, practicing, evaluating, or developing an understanding of systems or actions [10]. Simulators can be classified as low, medium, or high fidelity. Low-fidelity simulators are characterized by the minimal use of technology and are intended for skills training, while medium- and high-fidelity simulators allow for physiological responses to the interventions performed [11]. The high cost of veterinary simulators is an obstacle for educational institutions [12]. Providing training for clinical procedures using small animals with low-fidelity simulators has been developed and validated by researchers demonstrating how this approach offers an alternative with easy access and an excellent solution for learning [12,13]. By definition, self-confidence is the ability to trust oneself and one’s skills. [14,15]. The characteristics of a self-confident person are serenity and tranquility; even under pressure, he/she remains in emotional balance, as he/she is convinced about his/her ability to achieve [14,15]. During graduation, the development of self-confidence in the face of different clinical procedures is combined with the training provided by excellent professionals [15]. Neuroscientists have reported that positive emotions improve student learning and development. On the other hand, negative emotions result in a motivational decline that compromises memory and cognitive function [16]. Clinical simulation can reproduce a wide variety of adverse conditions within the routine, through which students can gain the necessary practice for mastering various techniques [8,9], it stimulates cognitive development as well as critical and reflective thinking, increasing students’ confidence in different situations, and improving their skills in a safe, risk-free environment [8,9].

Therefore, the aim of this study was to evaluate skill training using a low-fidelity simulator in the peripheral venipuncture procedure and examine the training’s influence on the *in vivo* procedure.

## Materials and Methods

### Ethical approval and informed consent

This study was approved under protocol No. 4008 by the Ethics Committees of Universidade do Oeste Paulista. All tutors authorized their animals to participate in the study through a consent document.

### Study period and location

The study was conducted from February to June 2018 at the Veterinary Hospital and the Laboratory of Skills of Universidade do Oeste Paulista.

### Study participants

The study involved 100 dogs attended to by the small animal medical clinic, 65 students in their 5^th^ year of undergraduate coursework in veterinary medicine, three veterinary doctors, and four external evaluators (previously trained). The study was divided into three different stages: (1) *In vivo* peripheral venipuncture (pre-training); (2) skill training with the low-fidelity simulator; and (3) peripheral venipuncture *in vivo* (post-training). A control group was not used in this study.

The checklists and self-confidence scale used were developed by three researchers in this field.

### Step 1: *In vivo* peripheral venous puncture (pre-training)

Initially, participants filled out a self-confidence scale on peripheral venipuncture procedure (Table-1) consisting of 18 items. During the 1^st^ week (7 days), under veterinary supervision, students collected blood from the dogs in the outpatient clinic. All students already had knowledge about the peripheral venipuncture procedure. In some cases, blood from the same animal was collected twice.

**Table-1:** Scale of self-confidence in the peripheral venous puncture procedure. Please refer to each item listed and identify how confident you are of being able to complete the task correctly. It is important to note that we are only evaluating your confidence level. Evaluate each item based on how confident you are about performing peripheral venous punctures in small animals:

S. No.	Item	1	2	3	4	5
1.	Assist in animal containment	( )	( )	( )	( )	( )
2.	Select blood vessel for collection	( )	( )	( )	( )	( )
3.	Opting or not for trichotomy	( )	( )	( )	( )	( )
4.	Select syringe or Vacutainer	( )	( )	( )	( )	( )
5.	Select needle gauge	( )	( )	( )	( )	( )
6.	Assemble material for collection	( )	( )	( )	( )	( )
7.	Perform antisepsis of the puncture site	( )	( )	( )	( )	( )
8.	Apply the tourniquet	( )	( )	( )	( )	( )
9.	Insert the needle into the skin	( )	( )	( )	( )	( )
10.	Find the chosen blood vessel	( )	( )	( )	( )	( )
11.	Remove the tourniquet	( )	( )	( )	( )	( )
12.	Decide the amount of material to be collected	( )	( )	( )	( )	( )
13.	Remove the needle from the skin	( )	( )	( )	( )	( )
14.	Compress puncture site to prevent bleeding	( )	( )	( )	( )	( )
15.	Homogenize the material (if necessary)	( )	( )	( )	( )	( )
16.	Fill the order correctly	( )	( )	( )	( )	( )
17.	Identify the material correctly	( )	( )	( )	( )	( )
18.	Forward to the lab	( )	( )	( )	( )	( )

1=Not confident, 2=Little confidence, 3=Confident, 4=Very confident, 5=Completely confident

During the blood collection process, an evaluator observed the procedure and filled out “Checklist 1 – Clinical evaluation of blood collection *in vivo*,” which contained 10 items related to blood collection (Table-2).

**Table-2:** Checklist 1 – Clinical evaluation of blood collection *in vivo*.

( ) Pre-training ( ) Post-training

Item	Description
1. Harvest spot	1. ( ) Cephalic 2. ( ) Jugular
2. Wearing gloves	1. ( ) Yes 2. ( ) No
3. Antisepsis	1. ( ) Yes 2. ( ) No
4. Number of harvest attempts	1. ( ) Single attempt 2. ( ) Multiple attempts 3. ( ) Waiver
5. Vacuum system use	1. ( ) Yes 2. ( ) No
6. Syringe and needle	1. ( ) Did not use syringe and needle 2. ( ) Used syringe and needle
7. Trichotomy	1. ( ) Collection with trichotomy 2. ( ) Collection without trichotomy
8. Complications	1. ( ) No complications 2. ( ) 1-3 complications 3. ( ) Above 3 complications
	Possible complications: ( ) Break blood vessel ( ) Hematoma ( ) Bleeding at the site ( ) None
9. Homogenization	1. ( ) Correct 2. ( ) Incorrect
10. Hemostasia of the puncture site	1. ( ) Adequate compression of the vessel (patient without bleeding) 2. ( ) Inappropriate vessel compression (patient with bleeding) 3. ( ) No compression of the vessel (patient with bleeding)

### Step 2: Skill training with the low-fidelity simulator

#### Simulator development

At the end of the last day of the 1^st^ week, students participated in a peripheral venipuncture skill training with a simulator developed from other studies [17,18].

**Figure-1:**
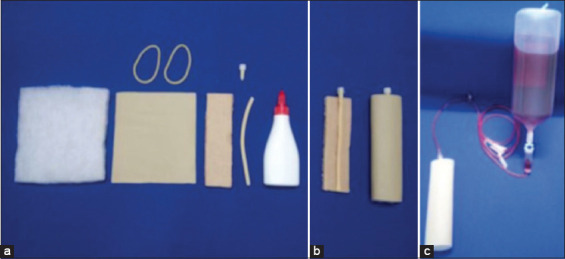
Materials used in the construction of the low-fidelity simulator for peripheral venipuncture training: (a) Acrylic blanket, piece of leather, latex elastic, cardboard, latex tube, non-needled end of 19G scalpel, and white glue; (b) simulator base ready to be covered with acrylic blanket and leather; low-fidelity simulator finalized; (c) simulator coupled with a disposable infusion set to a bottle of artificial blood prepared in advance.

**Figure-2:**
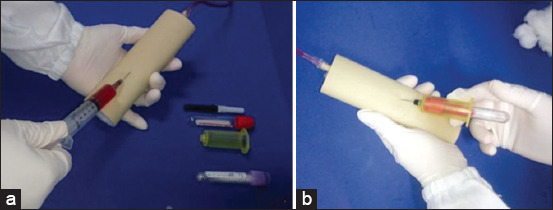
Low-fidelity simulator created for peripheral venipuncture training. (a) Demonstration of blood collection in the simulator using a syringe and needle; (b) demonstration of blood collection in the simulator with the vacuum tube system.

The developed simulator had low fidelity. For the creation of each simulator (Figures-1 and 2), a strip of cardboard (14×4 cm), latex tube (nº200 – with 14 cm of length), acrylic blanket (15×14 cm), piece of leather (15×14 cm), 19G non-needled scalpel end, two latex elastic bands (# 18), white glue, currant juice, empty physiological flasks, and a disposable infusion set were used.

A low-fidelity simulator was made available to each student. The total cost of each simulator was US$ 2.55.

The item was assembled by first closing one end of the latex tube using glue, and in the other, the non-needled end of the scalp was attached. This set, in turn, was glued onto the cardboard strip and wrapped in the acrylic blanket. The two elastic bands were attached to the leather to hold and cover the entire piece, simulating the animal’s skin.

The currant juice was used as artificial blood, and the empty physiological flasks were then filled with this liquid and coupled with the simulator through a disposable infusion set.

#### Skill training with the low-fidelity simulator

For each student, a simulator was made available for executing the skill training.

**Figure-3:**
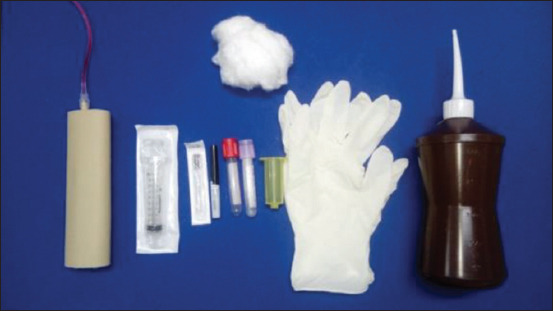
Materials available to students for simulated peripheral venipuncture training – complete low-fidelity simulator, 10 mL syringe, needle 25×0.70 mm, needle for Vacutainer 25×0.70 mm, biochemical tube, tube EDTA, yellow Vacutainer needle adapter, cotton, procedure gloves, and 70% alcohol.

Instructions for the materials used (Figure-3) in the procedure were given 10 mL syringe (Injex^®^, Brazil), 25×0.70 mm needle (Injex^®^, Brazil), 25×0.70 mm Vacutainer needle (BD^®^, Brazil), biochemical tube (BD®, Brazil), EDTA blood test tube (BD^®^, Brazil), yellow Vacutainer needle adapter (BD^®^, Brazil), cotton (DentalCremer^®^, Brazil), procedure gloves (Talge^®^, Brazil), and 70% alcohol (Prolink^®^, Brazil), as well for operating the simulator [2]. Next, the blood collection procedure using a syringe and needle and the vacuum system was demonstrated.

Students performed the peripheral venous puncture procedure, and each student was observed by an evaluator who completed “Checklist 2 – Evaluation of simulated training in peripheral venipuncture with the low-fidelity simulator,” comprised nine evaluation items (Table-3).

**Table-3:** Checklist 2 – Evaluation of simulated training in peripheral venipuncture with the low-fidelity simulator.

Item	
1. Wearing gloves	( ) Yes ( ) No
2. Site antisepsis	( ) Yes ( ) No
3. Ease of blood vessel location	( ) Yes ( ) No
4. Correctly positioning the bezel	( ) Yes ( ) No
5. Correctly transfer to the tube (blood count)	( ) Yes ( ) No
6. Easy to set up the vacuum system	( ) Yes ( ) No
7. Correct connection with the tube	( ) Yes ( ) No
8. Correct homogenization according to the tube	( ) Yes ( ) No
9. Puncture site hemostasis	( ) Yes ( ) No

The training time lasted 40 min; the student was not identified and was able to make several harvest attempts.

### Step 3: *In vivo* peripheral venous puncture (post-training)

During the 7 days following the simulator training, students collected blood *in vivo*. The self-confidence scale and Checklist 1 were repeated.

### Statistical analysis

For data analysis, the statistical environment R version 3.3.1 was used. In the first part, a descriptive analysis of the data was performed by calculating measures such as the average and standard deviation. Subsequently, the data set was divided into two groups, pre- and post-training. To test the normality of the data, the Kolmogorov–Smirnov test was performed followed by the Levene test for the homogeneity of the variances. It was verified that the data did not follow a normal distribution. The Wilcoxon non-parametric test was used for paired measurements, the same test was used for discrete variables where the normality test could not be applied. To evaluate the association between the nominal variables under study, the Fisher exact test was applied. For all analyses, a significance level of 5% was set [18].

## Results

In total, 130 blood samples were collected from the dogs (*in vivo*) at the outpatient clinic, and the peripheral venipuncture procedure was performed twice by 65 undergraduate students in veterinary medicine.

**Figure-4:**
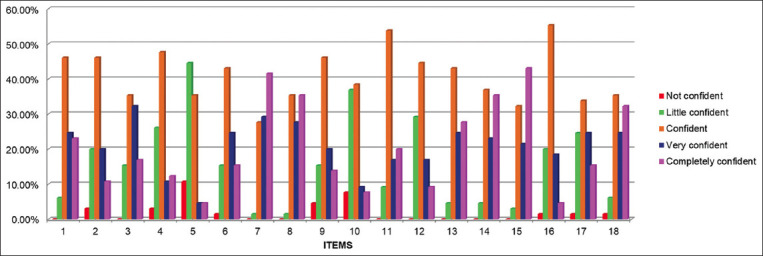
Histogram of the “Self-confidence scale of the peripheral venipuncture procedure” in the pre-training stage.

**Figure-5:**
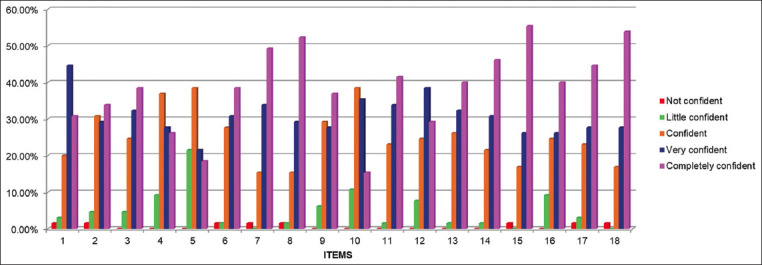
Histogram of the “Self-confidence scale of the peripheral venipuncture procedure” in the post-training stage.

In the self-confidence scale completed during the pre-training the items: Perform antisepsis at the puncture site and homogenize the material (if necessary) were the only ones where the students were completely confident (Figure-4). Observing the results of this scale in the post-training stage, most students were completely confident in 13 of the 18 items evaluated (Figure-5).

The results of Checklist 1 are described in Table-4. Statistically significant values were observed between the pre-training and post-training procedures for the items: Wearing gloves, the number of harvest attempts, complications, and puncture site hemostasis.

**Table-4:** Frequency result (%) of the items analyzed in “Checklist 1 – Clinical assessment of blood collection *in vivo*” in the pre- and post-training stages.

Item	Frequency (%) *n* = 130 collection		p-value

	Pre-training	Post-training	
Harvest spot			0.855
Cephalic	23 (35.33)	25 (38.47)	
Jugular	42 (64.67)	40 (61.53)	
Wearing gloves			<0.001*
Yes	13 (20.00)	39 (60.00)	
No	52 (80.00)	26 (40.00)	
Antisepsis			0.164
Yes	58 (89.23)	63 (96.93)	
No	7 (10.77)	2 (3.07)	
Number of harvest attempts			0.012*
A single collection attempt	31 (47.69)	46 (70.77)	
Multiple collection attempt	34 (52.31)	19 (29.23)	
Vacuum system use			0.763
Yes	7 (10.77)	5 (7.69)	
No	58 (89.23)	60 (92.31)	
Syringe and needle			0.763
Did not use syringe and needle	7 (10.77)	5 (7.69)	
Use syringe and needle	58 (89.23)	60 (92.31)	
Trichotomy			0.244
Collection with trichotomy	0 (0.00)	3 (4.62)	
Collection without trichotomy	65 (100)	62 (95.38)	
Complications			<0.001*
1-3 complications	29 (44.62)	7 (10.77)	
No complications	36 (55.38)	58 (89.23)	
Above 3 complications	0 (0.00)	0 (0.00)	
Homogenization			0.315
Correct	65 (100)	64 (98.46)	
Incorrect	0 (0.00)	1 (1.54)	
Puncture site hemostasis			0.001*
Adequate local compression time (patient did not present bleeding)	42 (64.62)	55 (84.62)	
Inadequate local compression time (patient had bleeding)	23 (35.38)	7 (10.77)	
No local compression (patient with large bleeding)	0 (0.00)	3 (4.61)	

* p<0.05

The frequency of different complication types occurred in 49 animals during the pre-training phase and only 9 during the post-training phase. Considering the same animal could simultaneously present more than 1 complication during the procedure, there was ample evidence that there was a significant association between the type of complication and the evaluation period (p<0.001) (Table-5).

**Table-5:** Result of frequency (%) of the item “possible complications” present in “Checklist 1 – Clinical evaluation of blood collection *in vivo*” in the pre- and post-training stages.

Complication	Frequency (%)		p-value

	Pre-training	Post-training	
Blood vessel rupture	25 (38.46)	2 (3.08)	<0.001*
Hematoma	8 (12.31)	0 (0)	
Bleeding at the spot	16 (24.62)	5 (7.69)	
None	36 (55.38)	58 (89.23)	

* p<0.05

The results for peripheral venipuncture training with a simulator demonstrated that there were differences in the frequencies of the items (Table-6).

**Table-6:** Result of frequency (%) of the items analyzed in “Checklist 2 – Evaluation of simulated training in peripheral venipuncture with the low-fidelity simulator” (n=65 students).

Item	Frequency (%)
Wearing gloves	
Yes	50 (76.92)
No	15 (23.08)
Site antisepsis	
Yes	63 (96.92)
No	2 (3.08)
Ease of blood vessel location	
Yes	40 (61.54)
No	25 (38.46)
Correctly positioning the bezel	
Yes	60 (92.31)
No	5 (7.69)
Correctly transfer to the tube (blood count)	
Yes	57 (87.69)
No	8 (12.31)
Easy to set up the vacuum system	
Yes	46 (70.77)
No	19 (29.23)
Correct connection with the tube	
Yes	58 (89.23)
No	7 (10.77)
Correct homogenization according to the tube	
Yes	63 (96.92)
No	2 (3.08)
Puncture site hemostasis	
Yes	51 (78.46)
No	14 (21.54)

## Discussion

Blood collection is indispensable in veterinary medicine. Many important diagnostic laboratory tests routinely require blood draws in medical practice with small animals. Therefore, a correct procedure for obtaining a quality blood sample is essential [2].

The positive influence of skills training on basic hospital procedures, as noted in this study, stresses including this learning tool in the curricular grids of different courses, as well as its use in professional recertification programs [19].

In the literature, skill training reports on peripheral venipuncture in humans demonstrate a better performance among students who were first exposed to the simulation and later harvested blood in real patients. These students described an improvement in the technique used, self-confidence, and emotional control during possible complications [19,20].

A realistic commercial model made of alpaca was used for training veterinary students to perform a jugular venipuncture [21]. However, students noted that the alpaca model did not move, hindering students’ actual procedure performance with a live animal [22]. Our low-fidelity model also lacked mobility, but this was not a limiting factor for students’ improved performance during the *in vivo* procedure.

Even low-fidelity models proved to be very efficient for student learning. One study developed a simulator of feline ovario salpingohysterectomy, which assessed the self-confidence levels of students who first underwent training on the model and then performed the procedure *in vivo* [16]. Their results showed increased self-confidence levels among students who trained on the model before surgery on real animals [16].

The data on self-confidence reinforce the importance of prior training for various procedures inherent to the veterinary clinic. In addition, low-fidelity models such as the one developed in this study and the one presented by other authors did not negatively interfere with developing participants’ self-confidence.

Regarding local complications in dogs after the peripheral venipuncture procedure, the most prevalent was blood vessel rupture followed by bleeding and, finally, hematoma formation.

After simulated training, 58 animals did not present any type of complication after blood collection. Clinical procedure complications can be attenuated when professionals are subjected to constant training, which enables acquiring knowledge about new techniques and developing new skills [22].

During the post-training period, the number of students who took blood samples with a single attempt was significantly higher (p=0.0012), regardless of the punctured site (jugular or cephalic vein), showing greater confidence among students when performing the procedure [23].

The syringe and needle choice was greater for blood collection compared to vacuum. In all likelihood, the lack of choice in vacuum collection was related to greater difficulty in assembling the system correctly, which demonstrates the importance of prior training for this procedure [23].

In terms of human health, there is growth in the integration of training for basic procedures in hospital routines [24].

These methodologies and tools are very well accepted by students and professionals. However, their positive impact on real-world patients in terms of reduced complications is secondary to poorly performed procedures [7,20].

The satisfactory results observed here during the post-training harvests corroborated other authors’ findings for different clinical or surgical veterinary procedures that used simulated training before implementation with real animals [25]. Training in dermatological examinations, laparoscopic techniques, and castration has proven to be highly efficient, enabling the students involved to develop skills and increase their self-confidence and safety [26,27].

In 2013, Eichel developed a horse head simulator using low-cost materials for training in drug administration through the jugular vein [25]. Students who underwent skill training on the simulator before performing the *in vivo* administration experienced a decreased procedure duration, less hesitation when starting to administer the drug, and reported that they were more apt and prepared for performing the procedure on the live animal [25].

Regarding the simulator developed in our research, the reproduction of the simplified anatomy was sufficient for the peripheral venipuncture procedure to be performed as many times as necessary, which corroborates with the findings of other authors. It is important to note, however, that further training on the patient is essential [28].

The simulator developed in this study enabled the development of students’ peripheral venipuncture skills at a low cost; namely, about US$ 2.55 or R$ 10.00 per simulator, which is close to the amount US$ 14.00 mentioned by another author [17]. These values are much lower than those available on the market, such as Surgireal’s Canine Leg Vascular Access® for $ 464.99 and UC Davis’s Canine Head and Neck Vascular Access Training model for $ 570.00 dollars, among others [1,17]. Thus, the low cost favors its use, since the high cost of simulators available on the market due to the high cost can limit training offered by institutions. Another important factor was being able to make and provide a simulator for each student, which streamlined the skill training and maintained their attention and focus.

Traditional teaching methods are increasingly being shown to be insufficient in training competent and self-confident professionals, which reflect on the quality of care and the safety of animal or human patients [29]. In a classic teaching environment, such as the classroom, students’ questions are more limited, making them passive agents in the learning context. This prevents both an analysis of team behavior and the possibility of immediate feedback in the face of diverse routine medical situations [29-31].

Compared to traditional teaching, a simulation promotes effective learning by providing a rich environment that encourages active and reflective student participation [29].

Teacher training is necessary to include clinical simulation in the undergraduate program, which leads to a break with traditional and conservative methodologies. It is necessary to discuss innovative methods within pedagogical projects. This should involve not only the inclusion of new technologies but also the use of new experiences that lead to the student’s autonomy, a process driven by the social context and the teacher’s role as a training agent [30,31].

There were some limitations in this study, such as not forming a control group. It was decided not to include a control group due to the fact that all students involved were in the last year of their coursework and already had previous knowledge of the procedure. The absence of movement in the simulator was also noted by some students as a negative model feature, as well as the lack of similarity with the real animal. However, its applicability was both satisfactory in view of the research objective and well accepted by the students, providing excellent results.

The development of low-fidelity simulators with low-cost materials must be a constant effort in veterinary medicine. The results of this research demonstrated that although a commercial simulator was not used, students’ learning was positively impacted by the developed tool. The same was also true of the results during the blood samples after the simulated training, which was also significant.

## Conclusion

Conducting skill training for peripheral venipuncture in animals using a low-fidelity simulator positively influences both students’ learning and their self-confidence in relation to *in vivo* harvesting, while also reducing the number of post-training complications, improving patients’ well-being.

The inclusion of teaching tools, such as clinical simulation and skill training linked to the disciplines present in the veterinary medicine curriculum, is fundamental for students’ skills development and competent training.

## Authors’ Contributions

DAFS and RMBN designed and guided the research steps. AAF, AEV, and AD collected the data. AMSS and CLS guided the storage of the collected samples and collection materials. GGSR conducted the statistical analysis of the data and created the graphs. All authors read and approved the final manuscript.
